# A Comparative Study of Neuroendocrine Heterogeneity in Small Cell Lung Cancer and Neuroblastoma

**DOI:** 10.1158/1541-7786.MCR-23-0002

**Published:** 2023-05-12

**Authors:** Ling Cai, Ralph J. DeBerardinis, Yang Xie, John D. Minna, Guanghua Xiao

**Affiliations:** 1 Quantitative Biomedical Research Center, Peter O'Donnell Jr. School of Public Health, UT Southwestern Medical Center, Dallas, Texas.; 2 Children's Research Institute, UT Southwestern Medical Center, Dallas, Texas.; 3 Simmons Comprehensive Cancer Center, UT Southwestern Medical Center, Dallas, Texas.; 4 Howard Hughes Medical Institute, University of Texas Southwestern Medical Center, Dallas, Texas.; 5 Department of Bioinformatics, University of Texas Southwestern Medical Center, Dallas, Texas.; 6 Hamon Center for Therapeutic Oncology Research, University of Texas Southwestern Medical Center, Dallas, Texas.; 7 Department of Pharmacology, UT Southwestern Medical Center, Dallas, Texas.; 8 Department of Internal Medicine, University of Texas Southwestern Medical Center, Dallas, Texas.

## Abstract

Lineage plasticity has long been documented in both small cell lung cancer (SCLC) and neuroblastoma, two clinically distinct neuroendocrine (NE) cancers. In this study, we quantified the NE features of cancer as NE scores and performed a systematic comparison of SCLC and neuroblastoma. We found neuroblastoma and SCLC cell lines have highly similar molecular profiles and shared therapeutic sensitivity. In addition, NE heterogeneity was observed at both the inter- and intra-cell line levels. Surprisingly, we did not find a significant association between NE scores and overall survival in SCLC or neuroblastoma. We described many shared and unique NE score–associated features between SCLC and neuroblastoma, including dysregulation of Myc oncogenes, alterations in protein expression, metabolism, drug resistance, and selective gene dependencies.

**Implications:**

Our work establishes a reference for molecular changes and vulnerabilities associated with NE to non-NE transdifferentiation through mutual validation of SCLC and neuroblastoma samples.

## Introduction

Small cell lung cancer (SCLC) and neuroblastoma are two very different cancer types with respect to their etiology, mutation spectrum/load, classification scheme, therapeutic strategy, and prognosis. SCLC, accounting for 13% of lung cancers, is predominantly found in heavy smokers, with almost ubiquitous comutation of *RB1* and *TP53*, and has a 5-year survival rate of 7% as the disease is highly metastatic. SCLC staging typically follows the two-stage classification convention established by the Veterans Affairs Lung Study Group (VALSG) in the 1980s. In the United States, two-thirds of patients with SCLC are diagnosed at the extensive stage, with cancers that have spread beyond the lung and nearby lymph nodes to other distant parts of the body. SCLC bears much resemblance to pulmonary neuroendocrine (NE) cells in their morphology and expression of NE markers (1), but studies from genetically engineered mouse models suggest that some SCLC may also arise from other lung cell types (2). Neuroblastoma, accounting for 6% of childhood cancers in the United States, is derived from sympathoadrenal progenitor cells within the neural crest (3), often develops in and around the adrenal gland, exhibits frequent genetic alterations in *MYCN* or *ALK*, and has a 5-year survival rate of 81%. Despite these differences, both SCLC and neuroblastoma are NE tumors, and NE markers are routinely used in IHC to facilitate the clinical diagnosis of both cancer types. As one of the “small round blue cell tumors” of childhood, undifferentiated neuroblastoma also highly resembles SCLC histologically.

Interestingly, the ability to transdifferentiate from the NE to non-NE lineage has been documented for both SCLC and neuroblastoma. Over 35 years ago, “classic” (NE) and “variant” (non-NE) SCLC were reported on the basis of distinct cellular morphologies and biochemical properties (4). In the recent decade, studies have shown that transdifferentiation of SCLC gives rise to intratumoral heterogeneity and mediates chemoresistance (5, 6). More recently, it was shown that REST, YAP, and NOTCH mediate NE transition in both SCLC and normal lung (7). For neuroblastoma, morphologically distinct cell types from cell lines established from the same patient tumor were observed over 50 years ago (8). Distinct biochemical properties and the ability to interconvert have been reported for isogenic cell subclones (9). In two more recent studies, the “sympathetic noradrenergic” (NE) and “neural crest cell-like” (non-NE; ref. 10), or “adrenergic” (NE) and “mesenchymal” (non-NE; ref. 11). Neuroblastoma cell states have been shown to exhibit distinct epigenetic and transcriptomic profiles. It has also been shown that NOTCH regulates transcription factor (TF) networks to drive NE transition in neuroblastoma and contribute to the development of chemoresistance in neuroblastoma (12). These independent studies converged on similar NOTCH-mediated mechanisms in NE lineage switch and suggest shared NE-associated properties across different cancer types. However, the extent of such similarity is still unclear. In this study, we reanalyzed the molecular and clinical data generated from SCLC and neuroblastoma cell lines and tumors to compare their associations with NE heterogeneity side-by-side, to reveal the concordance and idiosyncrasy in the landscape of NE state–associated features in both cancer types.

## Material and Methods

### Clustering of cell lines by multiomics, drug sensitivity, and dependency data

For the clustering of cell lines based on RNA sequencing (RNA-seq) data, we first conducted a principal component analysis for genes with a standard deviation larger than 0.4. The top 10 principal components accounted for 41% of the total variance and were used for hierarchical clustering. For the clustering of reverse phase protein array (RPPA) and metabolomics data, we used all available features and did not filter the input features or perform principal component analysis.

Compared with the molecular profiling data, functional screening data tend to be noisier due to variations in experimental design or lack of differential sensitivity among the cell lines (13). Therefore, for clustering dependency and drug data, we filter the input drug and dependency features by their consistency across multiple datasets. We collected nine compound screening datasets and three functional genomics datasets and conducted all possible combinations of pairwise correlations within the drug datasets and the dependency datasets, respectively. For example, for a specific drug profiled by four datasets, C(4,2) = 6 interstudy pairwise correlation would be available, with each interstudy pairwise correlation assessing the measurement consistency for the same set of cell lines in two datasets. We then summarized these interstudy pairwise Pearson correlations by meta-analysis to generate consistency measures for each compound and gene (13). For the clustering analysis in this study, we selected consistent dependency data features with *r* > 0.4, and for drug data, we selected consistent features with multiple comparison adjusted *P* values < 0.05. All hierarchical clustering was performed using Ward minimum variance method.

### NE score computation

The original SCLC NE signature based on microarray gene expression data was described by Zhang and colleagues (14). Here, we used the updated signature generated from RNA-seq expression (15). A quantitative NE score can be generated from an NE signature using the formula NE score = (correl NE − correl non-NE)/2, where correl NE (or non-NE) is the Pearson correlation between the expression of the 50 genes in the test sample and expression/weight of these genes in the NE (or non-NE) cell line group. This score ranges from −1 to +1, where a positive score predicts NE and a negative score predicts non-NE cell types. The higher the score in absolute value, the better the prediction.

### Comparison between bulk RNA-seq and single-cell RNA-seq data

Bulk RNA-seq data (CCLE_depMap_19Q1_TPM.csv) from cancer cell line encyclopedia (CCLE) and scRNA-seq data (GSE157220_CPM_data.txt.gz; ref. 16) downloaded from Gene Expression Omnibus (GEO) were used to compute the NE score for cell lines as well as single cells within cell lines using the above approach. For the single-cell RNA sequencing (scRNA-seq) data, the average NE score per cell line was calculated. A total of 191 cell lines were shared between the two datasets, including four SCLC and two neuroblastoma cell lines. Pearson correlation between the bulk RNA-seq NE scores and average scRNA-seq NE scores was used as a measure of agreement between the two profiling approaches.

### Data availability

#### Cell line datasets

Copy number, RNA-seq, miRNA, histone post translational modification (PTM), metabolomics, and RPPA data were downloaded from dependency map (DepMap). Compound sensitivity data for “CCLE” (17), “CTRP” (18), “GDSC1,” and “GDSC2” (19), “PRISM_1st,” and “PRISM_2nd” (20), and gene dependency data for demeter (RNAi; ref. 21) and achilles (ref. 22; CRISPR) were downloaded from DepMap and processed as described previously (13). The cell line names and compound names were unified, and the datasets were processed to ensure that the lower value in each dataset always corresponded to a higher sensitivity. The processed data, lists of consistent compounds, and dependencies were downloaded from https://lccl.shinyapps.io/FDCE/. The scRNA-seq data for cell lines were downloaded from the GEO repository GSE157220 (16).

#### Additional SCLC datasets

The following SCLC transcriptomic datasets “UTSW SCLC cell line,” “Drapkin_2018” [patient-derived xenograft (PDX)] (23), tumor datasets “Rudin_2012” (24), “George_2015” (25), “Jiang_2016” (26), and “Cai_2021” (15) were processed as described previously (15). The processed data are available in our previous publication (15). SCLC scRNA-seq data were downloaded from the HTAN portal (27).

#### Additional neuroblastoma datasets

In addition to the CCLE RNA-seq data, additional neuroblastoma cell line transcriptomic and associated sample phenotype data were downloaded from GEO using R package GEOquery (28) with the following accession numbers: GSE28019, GSE89413 (29), and GSE90683 (10). For neuroblastoma patient tumor datasets, we included two partially overlapped neuroblastoma datasets from Therapeutically Applicable Research to Generate Effective Treatments (TARGET; https://ocg.cancer.gov/programs/target) initiative, phs000467 (30). “TARGET_microarray” was downloaded from the TARGET Data Matrix, whereas “TARGET_RNA-seq” was downloaded from the UCSC Toil RNAseq Recompute Compendium (31). Additional neuroblastoma tumor datasets were downloaded from GEO with the following accession numbers: GSE120572 (32), GSE3446 (33), GSE19274 (34), GSE73517 (35), GSE85047 (36), GSE62564 (37), GSE16476 (38), and GSE3960 (39).

## Results

### Neuroblastoma and SCLC cell lines are molecularly similar

**Figure 1.**
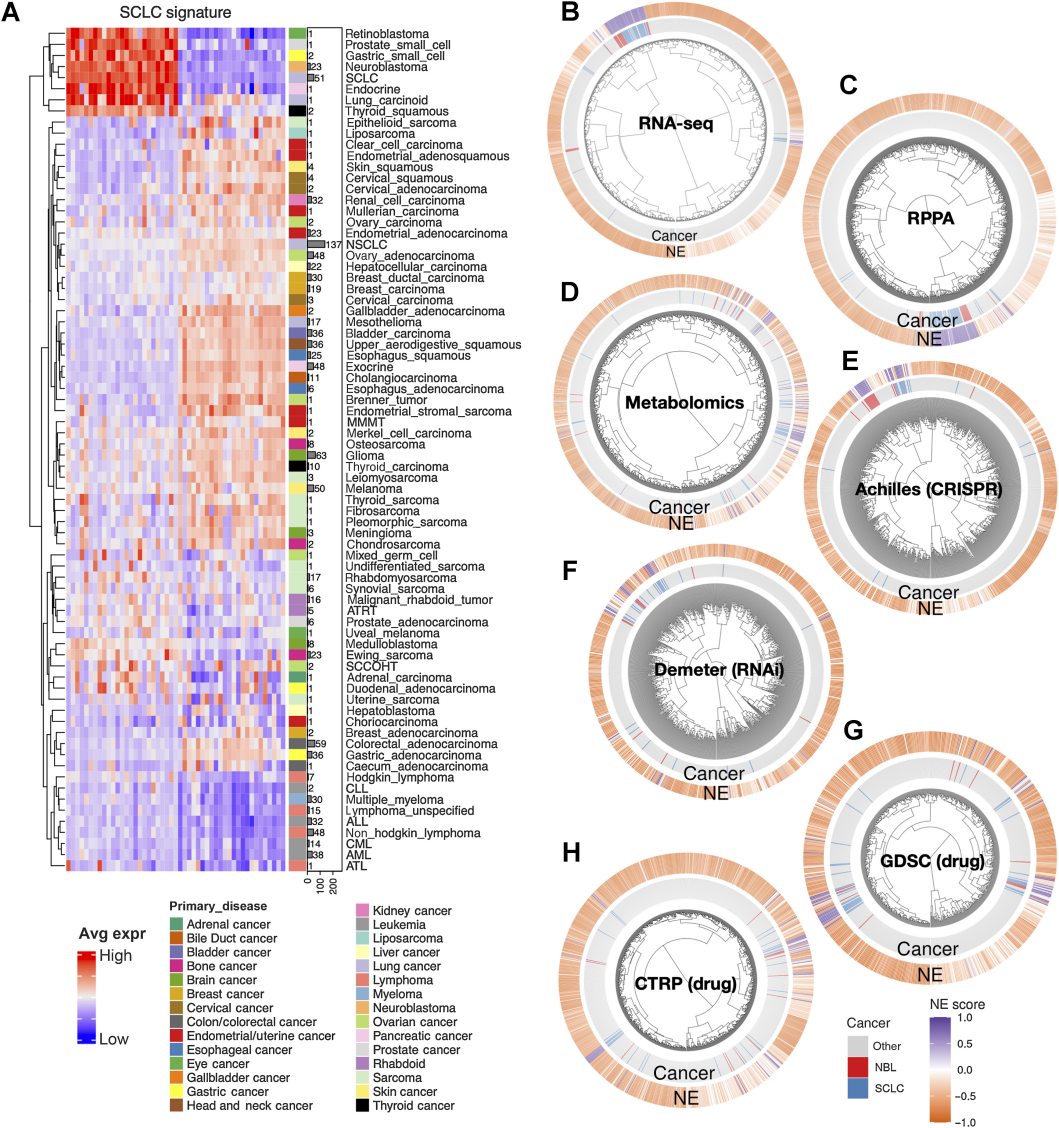
The molecular similarity between neuroblastoma and SCLC. **A,** SCLC NE signature gene expression across different cancer lineages. The expression of NE (left half) and non-NE (right half) genes were averaged by cancer lineages and plotted as a heat map. The number of cell lines per lineage are visualized as bars plotted right to the heat map. Note that SCLC and neuroblastoma are the two cancer types with the highest number of cell lines in the cluster with high expression of NE genes. **B**–**H,** Hierarchical clustering of cell lines by omics and functional screening datasets. The number of cell lines and number of features used for clustering are as follows: 1,165 cell lines by expression of 19,159 genes (**B**), 897 lines by 214 RPPA features (**C**), 926 lines by 225 metabolites (**D**), 688 lines by CRISPR effect score of 509 genes (**E**), 648 lines by RNAi effect score of 375 genes (**F**), 624 lines by 208 compounds from GDSC (**G**), and 794 lines by 168 compounds from CTRP (**H**). Note the clustering for RNA-seq data was based on the top 10 principal components, RPPA and metabolomics clusterings were based on all available features; dependency and drug clusterings were based on selected consistent features as previously summarized. Each leaf on the dendrogram represents a cell line. The inner rim right outside the dendrogram signifies the cancer lineage and the outer rim indicates the NE score of the cell line.

We have previously established an NE score calculation method for SCLC samples based on a gene expression signature generated from SCLC cell line transcriptomic data (14, 15). This method takes the expression data of 25 NE genes and 25 non-NE genes as inputs and assigns a score ranging from −1 (non-NE) to 1 (NE) to each sample. From the pan-cancer study CCLE/DepMap (40) RNA-seq dataset, we averaged the expression of these 50 genes by different cancer lineages and performed hierarchical clustering (Fig. 1A). Among cancer types in the subcluster with high expression of NE genes, SCLC and neuroblastoma had the highest number of cell lines in the CCLE collection. This allowed us to leverage the multidimensional profiling data from CCLE and DepMap for an in-depth comparison between SCLC and neuroblastoma. We computed NE scores for the pan-cancer cell lines and clustered the cell lines based on transcriptomic, functional proteomic (based on RPPAs), metabolomic, gene dependency, and drug sensitivity features (Fig. 1B–H). We observed tight clusters of SCLC and neuroblastoma cell lines with high NE scores in each clustering analysis. These results suggest that SCLC and neuroblastoma cell lines are highly similar in these molecular aspects.

### NE heterogeneity can be observed at inter- and intra-cell line levels for both SCLC and neuroblastoma

**Figure 2.**
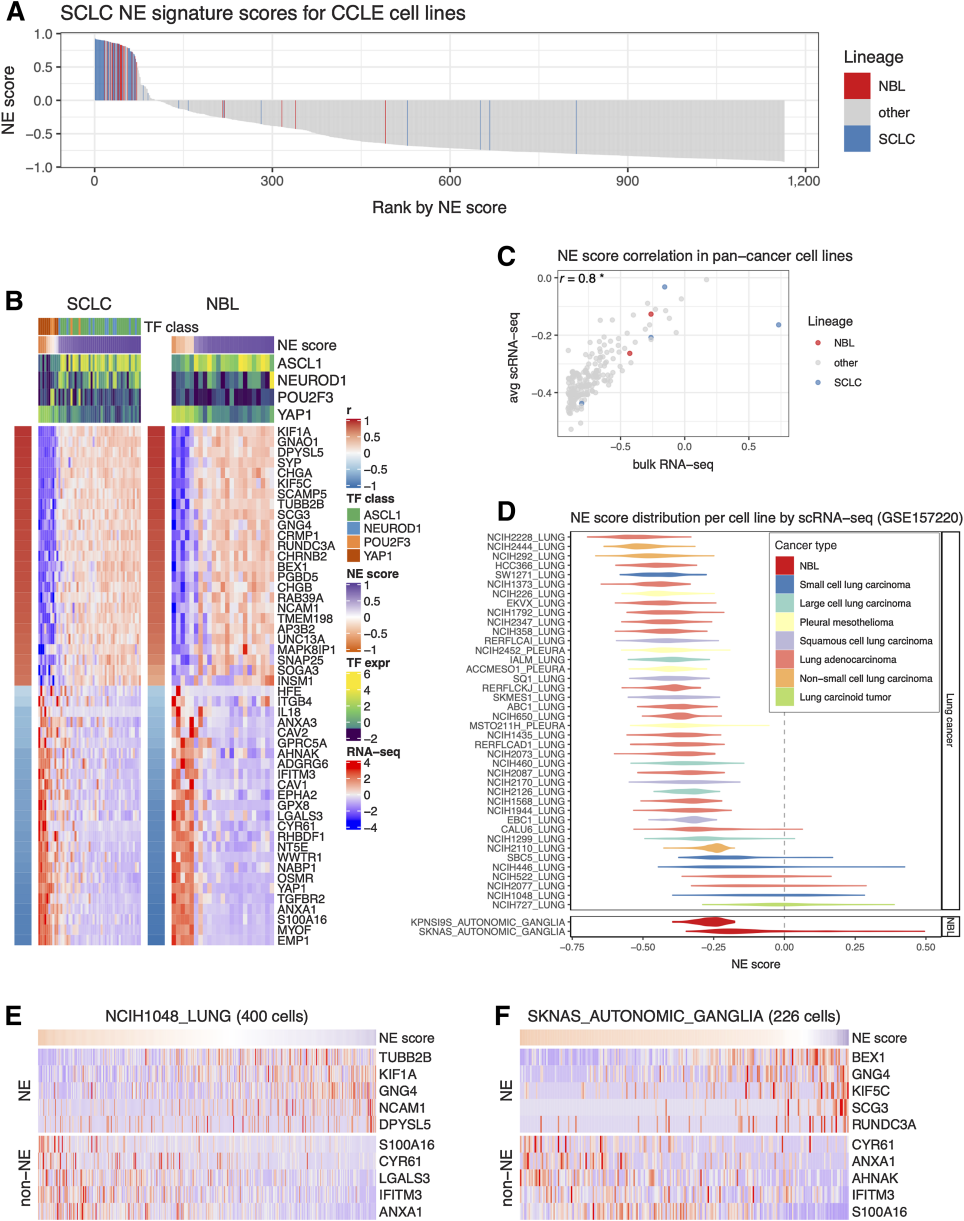
Inter- and intra-cell line NE heterogeneity. **A,** Inter-cell line NE heterogeneity. NE scores for CCLE pan-cancer cell lines were ranked from high to low. SCLC and neuroblastoma lines were highlighted by colors. Although most of the SCLC and neuroblastoma lines have high NE scores, a few of them also have low NE scores. **B,** Consistent gene expression pattern for SCLC NE signature genes observed for SCLC and neuroblastoma cell lines. Cell lines are in columns. Red/blue column left to the heat map annotates the correlation between the gene expression and NE score; the expression of SCLC driver TFs and NE scores was annotated above the heat map. SCLC lines were further classified into four TF classes. **C,** Average NE scores from scRNA-seq data align well with NE scores from bulk RNA-seq data for pan-cancer cell lines. **D,** Distribution of NE scores for lung cancer and neuroblastoma cell line–based scRNA-seq data. Intra-cell line NE heterogeneity. High- and low-NE score cells are found to coexist within the same SCLC cell line NCI-H1048 (**E**) or neuroblastoma cell line SKNAS (**F**). Single cells are in columns. Because of the high dropout rate of scRNA-seq data, only the top abundantly expressed genes are visualized.

We assessed NE heterogeneity in SCLC and neuroblastoma cell lines by ranking the cell lines in the CCLE panel based on their NE scores. While most of the SCLC and neuroblastoma cell lines had positive NE scores and were enriched in the top, a few cell lines had negative NE scores, revealing inter-cell line NE heterogeneity (Fig. 2A). We also examined the expression of SCLC NE score signature genes in SCLC and neuroblastoma cell lines. Although the signature was established in SCLC cell lines, it was also highly differentially expressed in neuroblastoma cell lines (Fig. 2B). Four key transcription factors (*ASCL1*, *NEUROD1*, *POU2F3*, and *YAP1*) have been proposed to define the four molecular subtypes of SCLC. We described their relationship with SCLC NE scores in our previous study (15). Although these transcription factors have not been used to classify neuroblastoma samples, when we examined their expression in neuroblastoma cell lines, we observed a pattern of segregation by NE score similar to SCLC—while high-NE-score neuroblastoma lines were found to have high expression of *ASCL1* or *NEUROD1*, low-NE-score neuroblastoma lines had high expression of *YAP1*. However, no neuroblastoma line was found to express high levels of *POU2F3*, a tuft cell regulator (ref. 41; Fig. 2B). These results suggest that similar transcriptional regulations are involved in driving NE heterogeneity in SCLC and neuroblastoma cell lines.

To explore NE heterogeneity at the intra-cell line level, we compared scRNA-seq–based average NE scores to bulk RNA-seq–based NE scores for 191 cell lines [using scRNA-seq data available for a panel of pan-cancer cell lines (16)] and found a strong correlation (Fig. 2C). We also observed that some SCLC and neuroblastoma cell lines had broader NE score distributions than others (Fig. 2D). Using scRNA-seq data from SCLC patient tumors, we further observed the coexistence of high-NE-score and low-NE-score SCLC cells within tumors that exhibited highly variable NE scores (Supplementary Fig. S1A–S1C). Similarly, upon examining NE and non-NE gene expression across single cells in the SCLC cell line NCI-H1048 and neuroblastoma cell line SKNAS, we also observed the coexistence of high-NE-score and low-NE-score cells within the same cell line (Fig. 2E and F). Notably, within these cell lines, cells with lower NE scores also had higher mesenchymal and IFN-response program scores, as annotated previously (16). In addition, low-NE-score cells from SCLC cell line NCI-H1048 had higher epithelial senescence–associated program scores, although this association was not statistically significant in neuroblastoma cell line SKNAS because neuroblastoma is not epithelial (Supplementary Fig. S1D). These findings indicate that the lineage heterogeneity observed in patient tumors is preserved both among cell lines and within individual cells from the same cell line.

### NE scores do not associate with overall survival in SCLC or neuroblastoma

**Figure 3.**
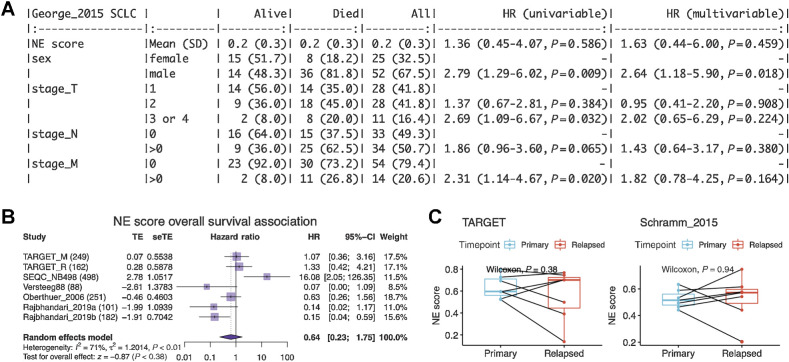
NE score is not associated with overall survival in SCLC or neuroblastoma. **A,** Survival association analysis for SCLC based on 79 patients from the George_2015 study. NE score is not significantly associated with overall survival in univariate Cox regression or a multivariate model controlling for sex and TNM stage. **B,** Meta-analysis for neuroblastoma based on seven studies and 1,531 patients. The result is also not statistically significant although significant results could be observed for individual studies, the trend was different. **C,** NE scores are not significantly altered in neuroblastoma relapsed samples. Paired samples from the same patients in two independent studies were compared.

Next, we tested whether NE scores were associated with disease outcomes in SCLC and neuroblastoma. As most patients with SCLC are diagnosed at an extensive stage, surgical resection of SCLC primary tumors is rare in practice. A recent study that profiled biopsied metastatic SCLC samples found no association between NE score and outcome (42). We also investigated the prognosis association in a previously published dataset generated from 81 surgically resected SCLC tumors, of which 30 are stage III–IV samples (25). We also did not find a significant association between the NE scores and overall patient survival (Fig. 3A). With multiple neuroblastoma tumor datasets available, we performed a meta-analysis to assess the association between NE scores and overall survival in neuroblastoma. We also did not observe a consistent and significant result (Fig. 3B). In the neuroblastoma datasets, we investigated, the previously reported prognostic factors—age, MYCN amplification, and INSS stage 4 disease—were consistently associated with worse overall survival (Supplementary Fig. S2A–S2C), but we did not observe a significant difference in NE scores in groups stratified by these factors (Supplementary Fig. S2D and S2F). A small effect size was observed for NE score difference by relapse/progression status (Supplementary Fig. S2G); however, when comparing paired naïve and relapse samples from the same patient in two independent neuroblastoma studies, we did not identify a statistically significant difference in NE scores (Fig. 3C). These findings suggest NE scores are not associated with prognosis in SCLC or neuroblastoma.

### Myc oncogenes are differentially activated by NE states in SCLC and neuroblastoma

**Figure 4.**
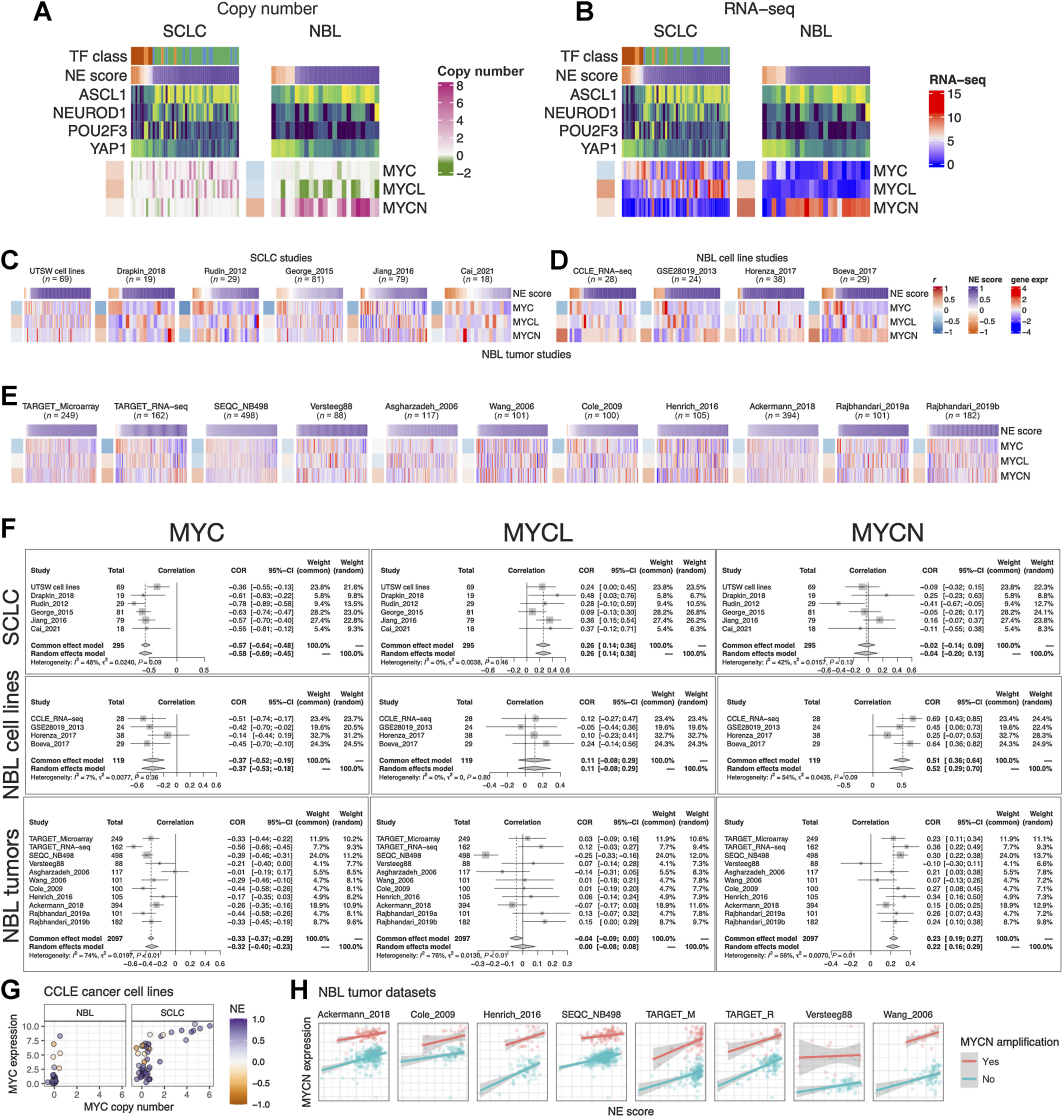
NE score association with members of the Myc oncogene family in SCLC and neuroblastoma. Copy number (**A**) and RNA expression (**B**) of Myc family genes in SCLC and neuroblastoma cell lines. Note that although MYC amplification was higher in the high-NE-score SCLC cell lines, its gene expression was higher in the low-NE-score cell lines for both SCLC and neuroblastoma lines. Frequent *MYCL* loss was found in neuroblastoma because *MYCL* is located in a frequently deleted region (chromosome 1p) in neuroblastoma. **C,** NE score versus Myc gene member expression in SCLC studies. “UTSW cell line” is a cell line dataset; “Drapkin_2018” is a PDX dataset; “Rudin_2012,” “George_2015,” “Jiang_2016,” and “Cai_2021” are all patient tumor datasets. NE score versus Myc gene member expression in neuroblastoma cell line datasets (**D**) and tumor datasets (**E**). Note that some of the same cell lines were profiled in multiple studies. **F,** Forest plots visualizing meta-analysis of NE score association with Myc family genes. *MYC* expression is consistently associated with lower NE scores in SCLC and neuroblastoma samples (left). *MYCL* expression positively correlates with NE scores in SCLC but not neuroblastoma samples (middle). *MYCN* expression positively correlates with NE scores in neuroblastoma but not SCLC samples. **G,** Relationship between *MYC* copy number and gene expression in neuroblastoma and SCLC cell lines. Note that *MYC* amplification is only observed in SCLC cell lines. **H,***MYCN* gene expression positively correlate with NE scores while controlling for *MYCN* amplification status in neuroblastoma patient tumors.

As members of the Myc oncogene family (*MYC*, *MYCN*, and *MYCL*) have been implicated in SCLC and neuroblastoma oncogenesis (43, 44), we attempted to dissect their relationship with the NE state. First, we examined copy-number alterations of Myc oncogenes (Fig. 4A). We found that *MYC* and *MYCL* were enriched in high-NE-score SCLC lines, whereas *MYCN* amplification was enriched in high-NE-score neuroblastoma lines. As MYCL is located on chromosome 1p, a frequently deleted region in neuroblastoma, *MYCL* loss appears to be frequent in neuroblastoma lines (Fig. 4A). Examination of the gene expression data showed that the patterns for *MYCL* and *MYCN* agreed well with the copy-number data (Fig. 4B). Having made these observations in cell lines, we further examined the transcriptomic data from multiple SCLC and neuroblastoma studies. For SCLC, we included our in-house cell line RNA-seq data (UTSW cell lines), PDX dataset (Drapkin_2018), and four tumor datasets (Fig. 4C). For neuroblastoma, we included three more cell line datasets along with the CCLE RNA-seq data (Fig. 4D) and assembled 11 tumor datasets (Fig. 4E). Meta-analyses with these datasets verified that the NE score associations with Myc oncogenes were consistent between multiple cell lines (Fig. 4B) and patient tumor datasets (Fig. 4F). Combined analysis of copy number, gene expression, and NE scores in the CCLE cell line dataset revealed upregulation of *MYC* expression in the low-NE-score lines without copy-number gain, suggesting the transcriptional activation of *MYC* expression in the non-NE state for both SCLC and neuroblastoma (Fig. 4G). We retrieved *MYCN* amplification status from eight neuroblastoma tumor datasets and assessed the association between NE score and *MYCN* expression while controlling for *MYCN* amplification (Fig. 4H). Much stronger associations were observed across multiple studies in this multivariate linear model (Supplementary Fig. S3), suggesting the transcriptional activation of *MYCN* expression in the NE state neuroblastoma tumors. In summary, SCLC and neuroblastoma exhibit not only differential copy-number gains but also differential transcriptional regulation for Myc family genes with regard to their NE status, with *MYC* transcriptionally upregulated in the non-NE state, *MYCL* preferentially amplified in high-NE-score SCLC, and *MYCN* preferentially amplified and transcriptionally upregulated in high-NE-score neuroblastoma.

### Consistent proteomic and metabolic changes are associated with NE-to-non-NE transition in SCLC and neuroblastoma

We performed NE score correlations with 12 sets of data from the CCLE/DepMap studies (Supplementary Tables S1 and S12). These include four sets of omics data (miRNA, histone PTM, RPPA, and metabolomics), six sets of compound screening data (CCLE, CTRP, GDSC1, GDSC2, PRISM_1ST, and PRISM_2nd), and two sets of gene dependency screening data (Demeter for RNAi and Achilles for CRISPR). The overall NE score association concordance was quite good for the omics datasets (Supplementary Fig. S4).

**Figure 5.**
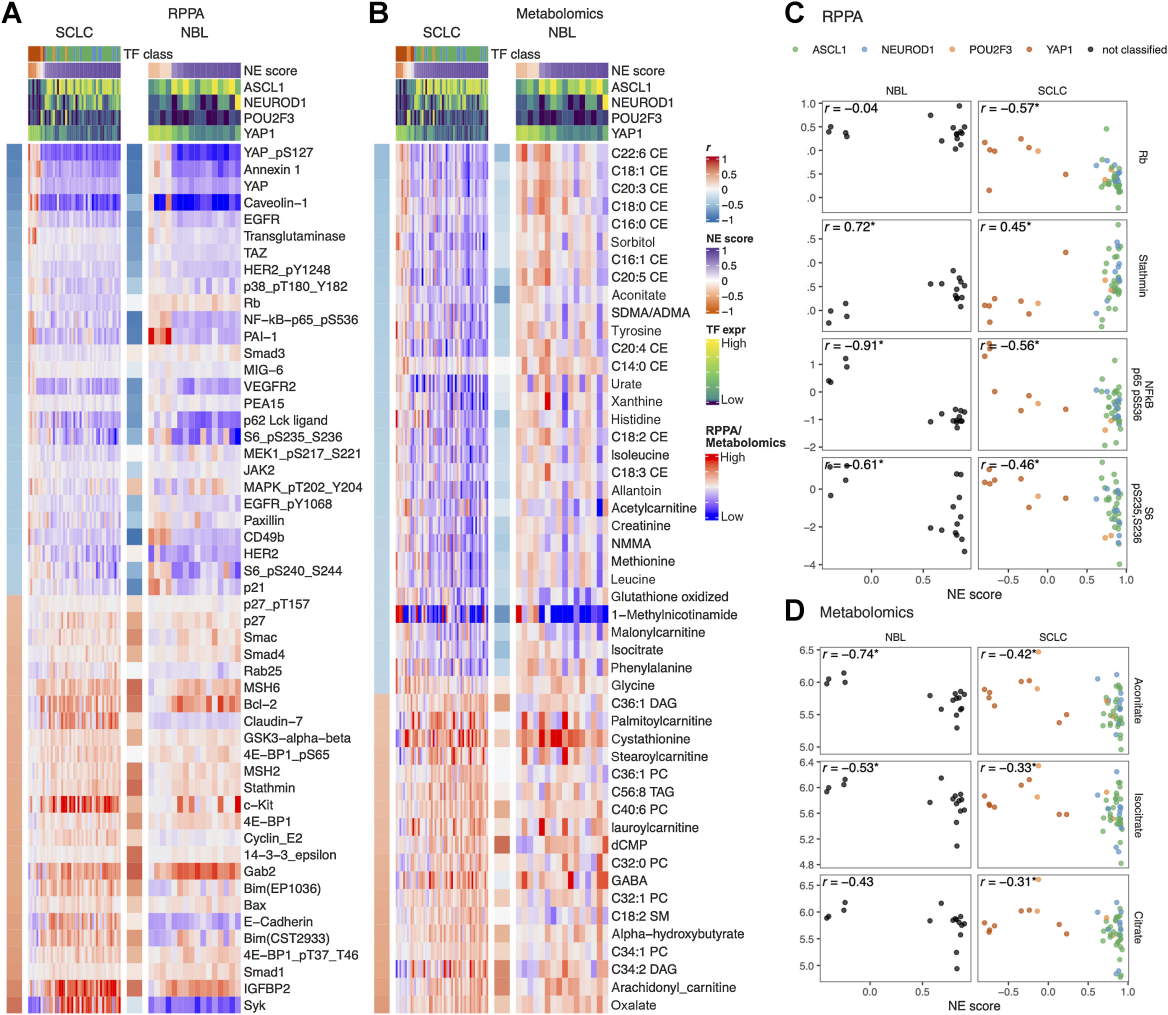
NE score–associated protein and metabolic features are largely consistent in SCLC and neuroblastoma cell lines. Heat maps visualizing the relationship between NE scores and selected functional proteomic feature (**A**) or metabolites (**B**). In each heat map, the left-side column denotes the Pearson correlation between the selected feature on the row and the NE score. The top colored rows denote NE scores and SCLC TF expression. The features were selected on the basis of NE score correlation from the SCLC cell lines, adjusted *P* value (*P*.adj) < 0.05 was used to select RPPA features and *P*.adj < 0.1 was used to select metabolic features. Note that although the selection was made from SCLC cell lines, a very similar pattern could be observed in neuroblastoma cell lines. Scatterplots visualizing the relationship between selected RPPA (**C**) and metabolic (**D**) features and NE scores in neuroblastoma and SCLC cell lines.

In Fig. 5A and B, we provide side-by-side comparison of the NE score-associated features in SCLC and NBL. In the RPPA associations (Fig. 5A), we found most of the NE score–associated features identified in SCLC cell lines could also be observed in neuroblastoma cell lines. Among the exceptions, Rb protein is decreased in the high-NE-score SCLC, leading to an increase in cyclin E2 but this was not observed in neuroblastoma lines (Fig. 5C), which could be explained by the frequent *RB1* loss that occurs in SCLC but not neuroblastoma. Although a previous study suggests *RB1* loss is highly enriched in YAP^off^ small-cell/neuro/NE cancer lineages (45), the absence of *RB1* mutation in neuroblastoma suggests the existence of an Rb-independent mechanism for YAP inactivation in neuroblastoma. Interestingly, in both SCLC and neuroblastoma, cyclin-dependent kinase–interacting protein/kinase inhibitory protein (CIP/KIP) p21 is upregulated in the low-NE-score lines whereas another CIP/KIP p27 is downregulated, suggesting that the NE state–specific cell-cycle regulators are still consistent in these two cancer types despite differences in the upstream Rb loss. In the low-NE-score lines of both SCLC and neuroblastoma, we observed higher levels of receptor tyrosine kinases and their phosphorylation (EGFR, EGFR_pY1068, HER2_pY1248, and VEGFR2), higher levels of Hippo signaling components (YAP, YAP_pS127, and TAZ), proinflammatory proteins (p62, NF-kB-p65_pS536, PAI-1, and annexin 1), ribosome biogenesis markers (S6_pS240_S244 and S6_pS235_S236), and cell adhesion proteins (paxillin and CD49b). In the high-NE-score lines of both SCLC and neuroblastoma, we found higher apoptotic machinery components (Smac, Bcl-2, Bim, and Bax), DNA repair proteins (MSH2 and MSH6), translation inhibitor 4E-BP1, and microtubule regulator Stathmin. Unique to SCLC, we observed higher epithelial junction proteins (Claudin-7 and E-cadherin) in the high-NE-score lines, these epithelial markers were however not expressed in the neuroblastoma lines (Fig. 5A). We also examined the metabolomic associations and observed similar consistency between SCLC and neuroblastoma lines (Fig. 5B). In particular, many cholesteryl esters were found to have higher levels in the low-NE-score SCLC lines; a weaker but similar trend was observed in neuroblastoma lines. We also found that both SCLC and neuroblastoma low-NE-score cell lines exhibited higher levels of citrate, aconitate, and isocitrate, three interconvertible metabolites, through the action of aconitase (Fig. 5B and D).

### Consistent and unique therapeutic vulnerabilities in NE and non-NE subtypes of SCLC and neuroblastoma

**Figure 6.**
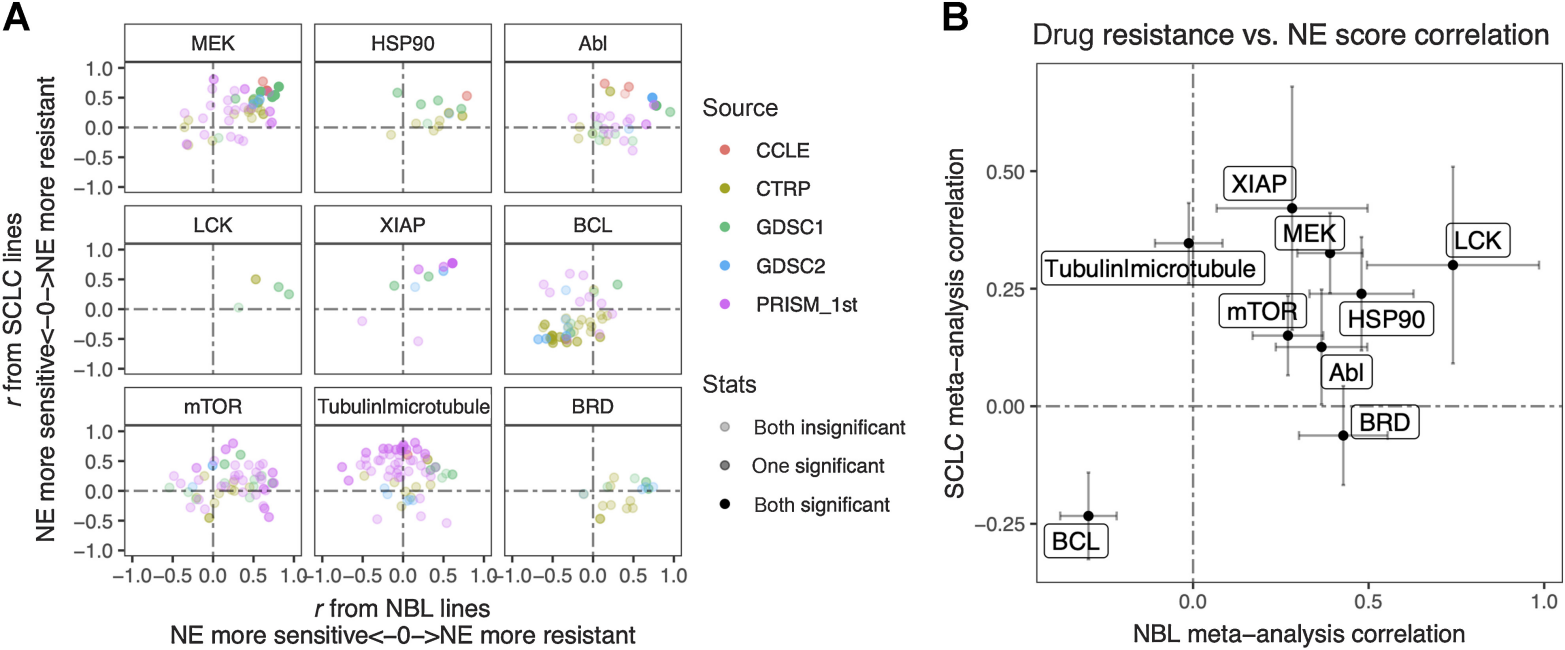
Similar and distinct NE score–associated therapeutic sensitivity in SCLC and neuroblastoma cell lines. **A,** Correlation between NE scores and therapeutic sensitivity for drugs with selected targets. Therapeutic sensitivity data were previously harmonized such that a higher value represents more resistance in each study. For each of the nine selected targets, all compounds with the same target were identified from multiple studies. Pearson correlation coefficient *r* from correlating compound data with NE scores were calculated for neuroblastoma lines (*x*-axis values) and SCLC lines (*y*-axis values), respectively and visualized as a scatter plot, with colors annotating the source of data, and transparency annotating the statistical significance. **B,** Meta-analysis–summarized correlation between drug therapeutic sensitivity and NE scores in neuroblastoma (*x*-axis) and SCLC (*y*-axis) cell lines. Note that high NE scores are associated with resistance to inhibitors of LCK, MEK, XIAP, mTOR, HSP90, and Abl, and sensitivity to BCL inhibitors. NE scores are associated with resistance to BRD inhibitors in neuroblastoma but not SCLC whereas microtubule inhibitors resistance correlates with high NE scores in SCLC but not neuroblastoma cell lines.

The SCLC versus neuroblastoma concordance for NE score–drug sensitivity associations was poorer than the omics data (Supplementary Fig. S4). We have previously demonstrated that drug screening data are more consistent for compounds directed against functionally important targets that are differentially expressed in a panel of cell lines (13). For many of the compounds included in the screens, their targets may not be functionally important in the small panel of cell lines tested, which may explain the overall lower consistency. We reviewed the results (Supplementary Tables S5 and S10) to identify the most consistent associations across the multiple compound screens. Nine classes of compounds with different mechanisms of action (MOA) were selected. For each MOA class, we compared the NE score associations for different compounds in neuroblastoma and SCLC (Fig. 6A). We also used meta-analysis to generate a summary correlation coefficient for each class of compounds from the SCLC and neuroblastoma assessments (Supplementary Figs. S5). We found that in both SCLC and neuroblastoma, cell lines with higher NE scores were more resistant to drugs that target MEK, mTOR, XIAP, LCK, HSP90, and Abl but were more sensitive to BCL inhibitors, which inhibit anti-apoptotic B-cell lymphoma-2 (Bcl-2) family of proteins. We also observed that higher NE scores were associated with resistance to microtubule inhibitors in SCLC, but not neuroblastoma cell lines, whereas higher NE scores were associated with resistance to bromodomain (BRD) inhibitors in neuroblastoma, but not SCLC lines (Fig. 6). Notably, although we identified differential therapeutic sensitivity within SCLC and neuroblastoma panels relative to their NE lineage, this does not tell us about the dynamic ranges of compound sensitivity in SCLC and neuroblastoma. In some cases, the dynamic range of compound sensitivity remains different between SCLC and neuroblastoma. For example, SCLC cell lines are the most resistant to MEK inhibitors, whereas neuroblastoma cell lines exhibit intermediate sensitivity over a broader range (Supplementary Fig. S6A–S6C). In other cases, we observed a similar overall sensitivity of SCLC and neuroblastoma cell lines. For example, both SCLC and neuroblastoma cell lines were more resistant to the HSP90 inhibitor 17-AAG, but more sensitive to the BCL inhibitor ABT-199, compared with the other cancer lineages (Supplementary Fig. S6D and S6E). In summary, our results revealed that the relative differential drug sensitivities associated with NE-to-non-NE transdifferentiation in SCLC and neuroblastoma were similar.

### Identification and comparison of SCLC and neuroblastoma-specific gene dependencies

**Figure 7.**
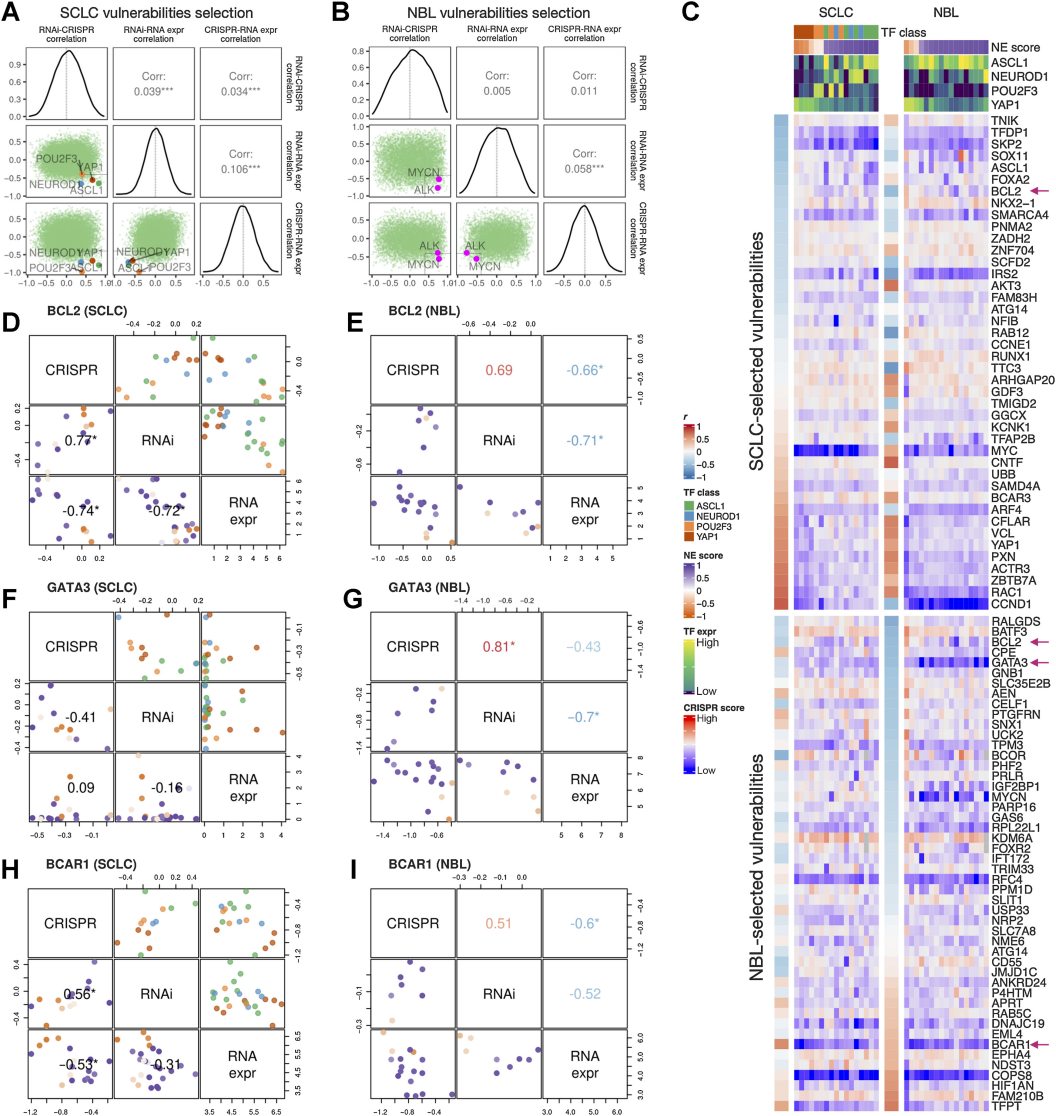
Similar and distinct NE score–associated gene dependencies in SCLC and neuroblastoma cell lines. Selection of SCLC (**A**) and neuroblastoma (**B**) vulnerabilities based on the consistency (positive correlation) between CRISPR and RNAi data, and anticorrelation between dependency data and gene expression data. Pearson correlation coefficients from RNAi-CRISPR (left), RNAi-RNA expr (middle), and CRISPR-RNA expr (right) correlations were computed for all genes. The distributions of these coefficients are plotted as diagonal panels; pairwise correlations among these three sets of correlation coefficients were visualized as scatter plots in the lower triangular panels and the Pearson correlation coefficients are printed in the upper triangular panels. The four SCLC subtype driver TFs and the neuroblastoma oncogenic driver MYCN all have high consistency between CRISPR and RNAi data and high anticorrelation between dependency data and gene expression data. Areas with *r* > 0.4 from RNAi-CRISPR correlation, and *r* < −0.4 from RNAi-RNA expr and CRISPR-RNAi correlation were demarcated by light gray squares. **C,** Correlation between NE scores and effect scores of selected dependencies in SCLC and neuroblastoma. The upper part of the heat map displays selected vulnerabilities for SCLC and was ordered by correlations between NE scores and the effect scores in SCLC cell lines; likewise, the lower part of the heat map displays selected vulnerabilities for neuroblastoma. Genes with magenta arrows are showcased in **D–I**. Cell lines are ordered by their NE scores and annotated with NE score and SCLC driver TF expression. **D–I,** Comparison of selected gene dependencies in SCLC and neuroblastoma. In each plot, variable names are shown in the diagonal boxes, and scatter plots display relationships between each pairwise combination of variables. Lower triangular plots are colored by NE scores whereas upper triangular plots for SCLC figures are colored by TF classes. Pearson correlation coefficients are provided in lower triangular boxes for SCLC and upper triangular boxes for neuroblastoma. Refer to legends in **C** for color annotations.

We observed very poor overall concordance between the RNAi and CRISPR dependency data for their association with NE scores in SCLC and neuroblastoma (Supplementary Fig. S4). We rationalized that this is because most genes were not selectively essential in the relatively small panel of SCLC or neuroblastoma cell lines assessed. Hence, we adopted a set of criteria for selecting cancer-specific gene dependencies. We looked for genes with RNAi versus CRISPR gene effect scores positively correlated, as an indication of high reproducibility from independent dependency screening experiments, as well as negative correlations between RNAi or CRISPR gene effect scores and RNA-seq expression data on the premise that genes of selective functional importance are more highly expressed in the cells that depend on them, such that these cells also have more negative gene effect scores that indicate higher dependence. These measures from the SCLC and neuroblastoma panels were assembled to prioritize the SCLC-specific vulnerabilities (Supplementary Table S13). Indeed, when we examined these correlations, the known SCLC subtype drivers and the most common neuroblastoma driver genes all met this set of criteria (Fig. 7A and B). We further closely examined genes with high RNAi versus CRISPR correlation, and high anticorrelations between RNA expression and the gene effect scores as selected vulnerabilities (Fig. 7C). Among the SCLC-selected vulnerabilities, along with ASCL1, we found several other NE lineage transcription factors (*SOX11*, *FOXA2*, *NKX2-1*) were more selectively essential for high-NE-score cell lines, whereas several genes involved in cell adhesion and motility (*VCL*, *PXN*, *ACTR3*, and *RAC1*) were found to be more selectively essential for low-NE-score cell lines; we also found genes frequently amplified in SCLC (*IRS2*, *CCNE1*, and *NFIB*; ref. 46) although these genes do not have gene effect scores significantly correlated with NE scores. Interestingly, among these SCLC-selected vulnerabilities, we also identified genes that are well characterized for their roles in neuroblastoma, such as the ciliary neurotrophic factor *CNTF* (47) and S-phase kinase-associated protein 2 *(SKP2*; ref. 48). Among the very few vulnerabilities selected from both SCLC and neuroblastoma, we identified *BCL2*, a well-characterized gene in both cancer types. Consistent with our observation in the therapeutic sensitivity analysis, high-NE-score cell lines from both SCLC and neuroblastoma were more sensitive to *BCL2* depletion (Fig. 7D and E). As only nine neuroblastoma cell lines were included in the RNAi dependency screen, the reliability of our neuroblastoma-selected vulnerabilities might have been undermined by the underpowered input datasets. Nevertheless, we were able to identify a few genes known to be important for neuroblastoma, such as GATA binding protein 3 *GATA3* (49), complement decay-accelerating factor *CD55* (50), forkhead box R2 *FOXR2* (51), and breast cancer antiestrogen resistance protein 1 *BCAR1*/p130Cas (52). Among these, selective essentiality for *GATA3* was only observed for high-NE-score neuroblastoma cell lines but not SCLC cell lines (Fig. 7F and G), whereas *BCAR1* appears to be a shared vulnerability for low-NE-score cell lines in both neuroblastoma and SCLC (Fig. 7H and I). Overall, we observed unique and shared gene dependencies between SCLC and neuroblastoma cell lines, some of which also exhibited NE/non–NE lineage–specific selectivity.

### Validations in additional cancer types

Given the distinct differences in etiology, risk factors, and molecular mechanisms between SCLC and neuroblastoma, we sought to validate our findings by analyzing cell lines from other cancer types. To this end, we identified lineage subtypes with at least two cell lines that exhibited positive NE scores based on transcriptomic data (Supplementary Fig. S7A) and selected cell lines from medulloblastoma, prostate adenocarcinoma, Ewing sarcoma, and non–small cell lung cancer (NSCLC) for further investigation.

We confirmed that the original SCLC NE signature genes were differentially expressed by NE subtype in these cell lines (Supplementary Fig. S7B). We also examined the RPPA and metabolomics features that were associated with NE scores in SCLC, finding good agreement with these features in the four additional cancer types (Supplementary Fig. S7C and S7D). Furthermore, we explored the relationship between Myc gene family members’ copy number and RNA expression and NE scores in the four cancer types. However, we observed no consistent pattern (Supplementary Fig. S7E). Notably, while c-Myc expression was anticorrelated with NE scores in SCLC and neuroblastoma, we found a strong positive correlation in medulloblastoma.

Because of the very limited number of cell lines available for drug sensitivity profiling in medulloblastoma, prostate adenocarcinoma, and Ewing sarcoma, we only compared SCLC and NSCLC for their NE score–associated drug sensitivity. Our results indicated agreement for MEK and BCL inhibitors (Supplementary Fig. S7F). However, this finding is not entirely robust, as NSCLC has few NE-positive cell lines, and even fewer were profiled for drug sensitivity.

## Discussion

Different cancers of the NE lineage have historically been investigated as separate entities, owing to their distinct clinical presentations. The common Notch-mediated NE lineage plasticity and the adjacency of SCLC and neuroblastoma in cancer cell line clustering by multi-omics datasets (Fig. 1) prompted us to perform a systematic comparison of these two cancer types. In this article, we identified numerous common molecular associations with NE states in both cancer types. Most of the proteomic and metabolic features observed to associate with NE states in SCLC could be validated in neuroblastoma (Fig. 5). NE score–associated transcriptomes are also highly similar between SCLC and neuroblastoma. We previously reported cell-autonomous immune gene repression in SCLC and pulmonary NE cells in the NE state and transdifferentiation into the non-NE lineage releases the repression of immune genes (15). Recently, similar observations have been reported for neuroblastoma (53). Besides immune genes, many other genes also are differentially expressed by NE status in both SCLC and neuroblastoma. Although our omics analyses in this study do not include large-scale transcriptomics comparison, this topic is explored in greater depth in a companion article (54).

In the cell line cluster generated by RNA-seq, RPPA, metabolomics, drug sensitivity, and gene dependency data, we observed neuroblastoma and SCLC lines cluster tighter with each other in RNA-seq and RPPA data (Fig. 1B–H). There may be several potential reasons. It is possible that while gene and protein expression patterns are hardwired by lineage specificity, the cell metabolism and functional liabilities are subjected to many additional feedback regulations. For example, chromosome instability may not be related to NE transdifferentiation, but the resulting replication stress could modulate nucleotide biosynthesis metabolism (55) and alter a cell's response to DNA-damaging drugs. Another contributing factor may be the insufficient coverage of metabolome and the drug targets of the drug screening panels may not adequately capture the functions differentially regulated by NE transdifferentiation. It is also worth noting that drug and gene dependency datasets are generally noisier than molecular profiling data (4), which may explain their poor NE score correlation agreement between SCLC and neuroblastoma (Supplementary Fig. S4), as well as their less robust clustering of the SCLC and neuroblastoma cell lines.

Our investigation of Myc family members in Fig. 4 revealed that *MYCN* amplification is enriched in high-NE-score neuroblastoma cell lines, *MYCL* amplification is enriched in high-NE-score SCLC cell lines, whereas increased *MYC* gene expression is observed in low-NE-score cell lines and tumor samples of both SCLC and neuroblastoma. Interestingly, MYCN has been shown to drive NE prostate cancer initiation (56), as it can epigenetically activate neural lineage gene expression in prostate cancer (57). A similar mechanism may also apply to neuroblastoma, where the dependence on *MYCN* to epigenetically sustain NE lineage could explain the high *MYCN* levels observed in samples with high NE scores. On the other hand, in a SCLC mouse model, it has been shown that c-Myc can activate Notch to drive the loss of NE fate (58). Upon c-Myc activation, SCLC cells undergo a transition from Ascl1-positive state to Neurod1-positive state (59), and eventually to Yap1-positive state (58). In another mouse model of SCLC, it has been shown that loss of *Ascl1* can revert SCLC to a more neural crest–like fate (60). In neuroblastoma, while the ASCL1-high adrenergic-type (NE) cells are committed to the adrenergic lineage, the ASCL1-low mesenchymal-type (non-NE) neuroblastoma cells are also known to resemble neural crest–derived precursor cells (12). Therefore, the interplay between Myc family members, ASCL1, Notch signaling, and other factors in regulating NE plasticity in cancer may be reflecting their roles in driving cell fate toward NE or non-NE lineage during normal tissue development. However, whether these findings can be generalized to other cancer types remained to be verified. As shown in Supplementary Fig. S7E, some medulloblastoma cell lines with high MYC expression still exhibit high NE scores, indicating that c-Myc activation does not always result in the loss of NE fate. In addition, loss of NE fate may occur upon Notch activation regardless of c-Myc status (7). Furthermore, it is important to note that different types of cancer may involve distinct factors in the regulation of NE transdifferentiation. For example, NE prostate cancer driven by MYCN is highly dependent on Rb deletion (61), while high *MYCN* expression in neuroblastoma does not coincide with Rb deletion. Therefore, the specific factors that collaborate to regulate NE fate in different cancer types may impact the generalizability of MYC-dependent NE fate modulation. Further research is needed to fully understand the role of Myc genes and others in the complex regulation of NE transdifferentiation in different types of cancer.

Our comparison of SCLC and neuroblastoma therapeutic vulnerabilities revealed similar NE score associations for several classes of compounds with shared MOA. Interestingly, individual studies in SCLC or neuroblastoma have also reported many of these associations, such as MEK inhibitors for neuroblastoma (62), HSP90 inhibitors for SCLC (63) and neuroblastoma (64), and BCL2 inhibitors for SCLC (65) and neuroblastoma (66). We also identified cancer-unique vulnerabilities that have been previously reported, such as BRD inhibitors and GATA3 essentiality for neuroblastoma (67, 68). Our findings suggest that NE plasticity may serve as a venue for therapy resistance in both SCLC and neuroblastoma for such drugs, as long-term monotherapy targeting cells of one lineage may create a selective pressure that shifts the population toward the other lineage. Importantly, our systematic investigation has mapped out the lineage-specific vulnerabilities in the NE and non-NE states. Coupled with our observation that high-NE-score and low-NE-score cells can coexist within the same SCLC or neuroblastoma cell line (Fig. 2), it would be interesting for future work to devise combinatorial therapies with drugs that target both NE and non-NE states and compare the efficacy with monotherapy using cell lines or cell line–derived xenografts as preclinical models. Although we did not experimentally validate the NE lineage–specific therapeutic vulnerabilities identified in this study, we have used five compound screening datasets and two gene essentiality screening datasets to choose features based on agreement across multiple datasets to maximize result reliability. Therefore, we believe that the generated results are credible and offer valuable insights for future work.

Compared with the omics analyses, relatively fewer similarities were observed at the functional liability levels. Several reasons might explain this discrepancy. First, functional data are much noisier than -omics data (13). Second, fewer SCLC and neuroblastoma cell lines were included in the functional screening datasets and this compromised the statistical power for target discovery (Supplementary Fig. S4). As our concordance-based approach requires examining data from common cell lines between two datasets, this further reduces the available sample size for analysis. Finally, the unique vulnerabilities for SCLC and neuroblastoma may stem from cancer drivers that act orthogonally to NE status. One such example is *NFIB*, which has been characterized as a metastatic promoter in SCLC (69).

In summary, our study provides a comprehensive molecular reference for features and vulnerabilities associated with NE-to-non-NE lineage transitions in SCLC and neuroblastoma. We also identified unique features that require further investigation in the context of each cancer type. While our focus was on SCLC and neuroblastoma, which are known for their NE-to-non-NE transitions, we discovered that many molecular features associated with NE scores in SCLC are also relevant to other cancers, such as prostate cancer and NSCLC, which exhibit non-NE-to-NE transitions to develop resistance to therapy (see Supplementary Fig. S7). Overall, our findings can guide the development of combinatorial therapies targeting lineage plasticity in SCLC, neuroblastoma, and other cancers that display NE heterogeneity.

## Supplementary Material

Figure S1 Intratumoral NE heterogeneity in SCLC patient tumors and intra-cell line NE heterogeneity in SCLC and NBL cell lines.

Figure S2 Known NBL prognostic factors consistently associate with outcome across different NBL studies but not NE scores.

Figure S3 Controlling for MYCN amplification status increases the statistical significance of NE score vs. MYCN expression association.

Figure S4 SCLC vs. NBL concordance of NE score association with omics, drug sensitivity, and dependency data.

Figure S5 A meta-analysis example that summarizes the association between MEK inhibitor resistance and NE scores.

Figure S6 Examples of different and similar compound sensitivity dynamic ranges in SCLC and NBL.

Figure S7 Comparing NE score-associated features between SCLC and other cancer types.
