# Supplementary material for: A Comparative Study of Neuroendocrine Heterogeneity in Small Cell Lung Cancer and Neuroblastoma
Source: Mol Cancer Res. 2023 May 12;21(8):795–807. doi: 10.1158/1541-7786.MCR-23-0002 (PMC10390888; doi:10.1158/1541-7786.MCR-23-0002)
Supplement: Figure S1 — Intratumoral NE heterogeneity in SCLC patient tumors and intra-cell line NE heterogeneity in SCLC and NBL cell lines. [file mcr-23-0002_figure_s1_suppsf1.pdf]

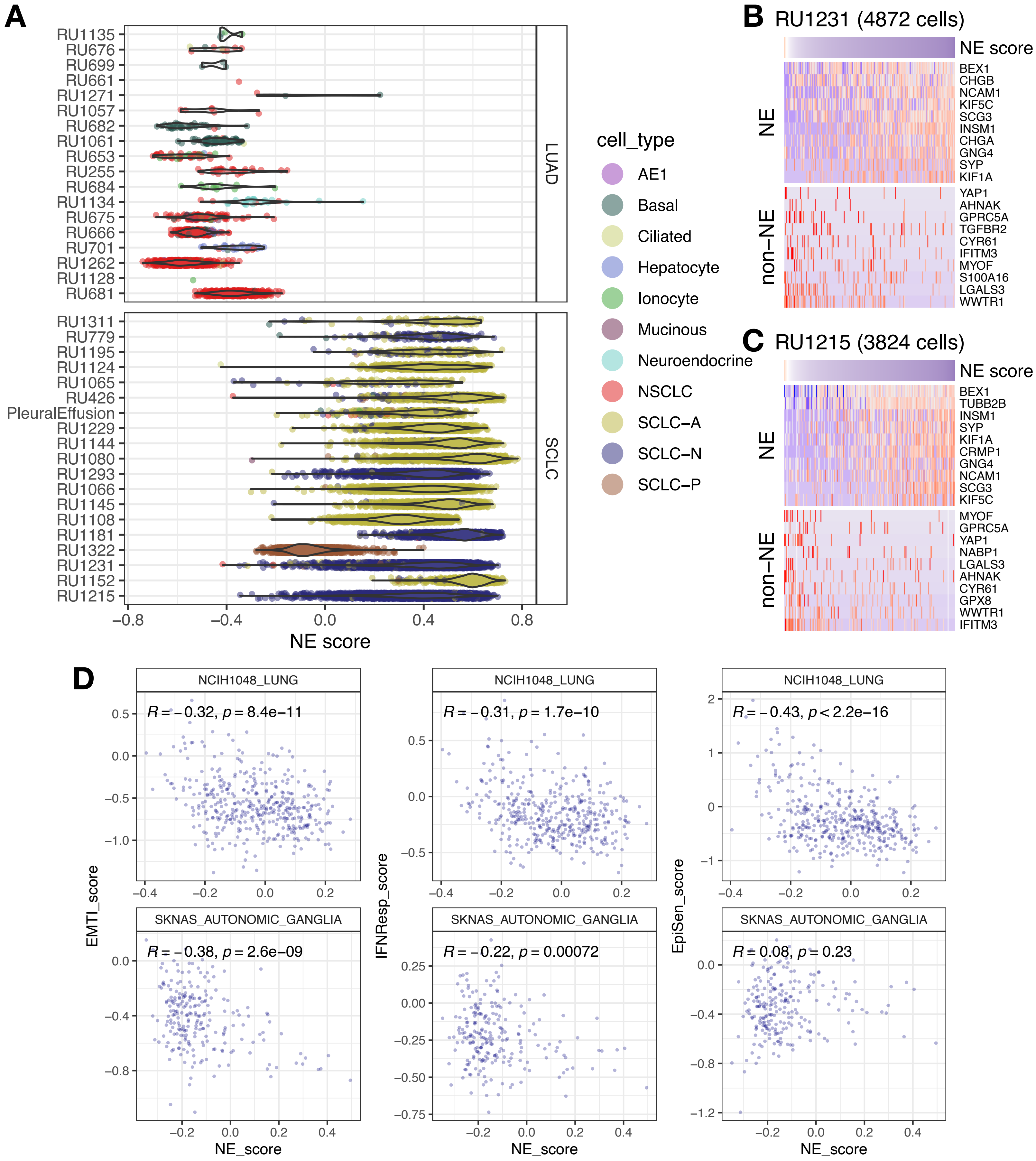

Figure S1. Intratumoral NE heterogeneity in SCLC patient tumors and intra-cell line NE heterogeneity in SCLC and NBL cell lines

A. Distribution of NE scores for lung cancer patient tumors from HTAN datasets. Sample names are labeled on the y-axis. Each dot represents a cell within the sample and is colored by the cell type annotated by the original study, violin plot is overlaid to visualize the overall distribution. Note that two SCLC-N tumors (RU1231 and RU1215) have broad NE score distributions. **B-C**. High- and low- NE score tumor cells are found to coexist within the same SCLC tumor. Single cells are in columns. Due to the high dropout rate of scRNA-seq data, only the top abundantly expressed genes are visualized. **D**, NE score (x-axis) correlation with EMT score, interferon response program score, and epithelial senescence program score (y-axis) in single cells of SCLC cell line NCIH1048 and NBL cell line SKNAS from Kinker et al.'s study.
