# Supplementary material for: A Comparative Study of Neuroendocrine Heterogeneity in Small Cell Lung Cancer and Neuroblastoma
Source: Mol Cancer Res. 2023 May 12;21(8):795–807. doi: 10.1158/1541-7786.MCR-23-0002 (PMC10390888; doi:10.1158/1541-7786.MCR-23-0002)
Supplement: Figure S2 — Known NBL prognostic factors consistently associate with outcome across different NBL studies but not NE scores. [file mcr-23-0002_figure_s2_suppsf2.pdf]

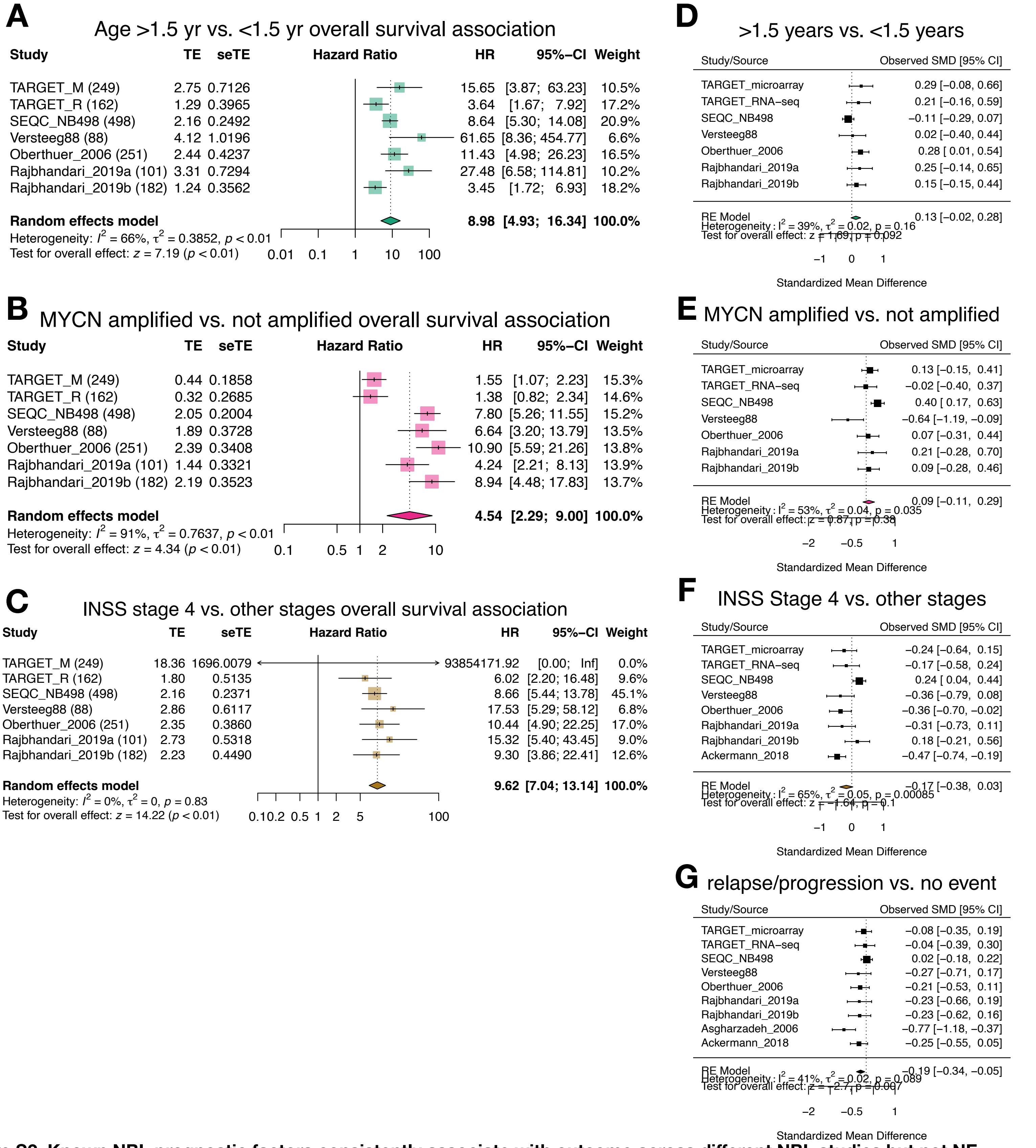

Figure S2. Known NBL prognostic factors consistently associate with outcome across different NBL studies but not NE scores

A-C. Age at diagnosis (**A**), MYCN amplification (**B**), and INSS stage 4 (**C**) are significantly associated with worse outcome. **D-G.** Meta-analyses of NE scores standardized mean difference between groups stratified by different clinical features. Age at diagnosis (**D**), MYCN amplification (**E**) and INSS stage 4 (**F**) are not significantly associated with NE scores. Lower NE scores are associated with relapse (**G**). Note that although one out of the nine studies showed a significant result, the overall result from the meta-analysis is statistically significant.
