# Supplementary material for: A Comparative Study of Neuroendocrine Heterogeneity in Small Cell Lung Cancer and Neuroblastoma
Source: Mol Cancer Res. 2023 May 12;21(8):795–807. doi: 10.1158/1541-7786.MCR-23-0002 (PMC10390888; doi:10.1158/1541-7786.MCR-23-0002)
Supplement: Figure S3 — Controlling for MYCN amplification status increases the statistical significance of NE score vs. MYCN expression association. [file mcr-23-0002_figure_s3_suppsf3.pdf]

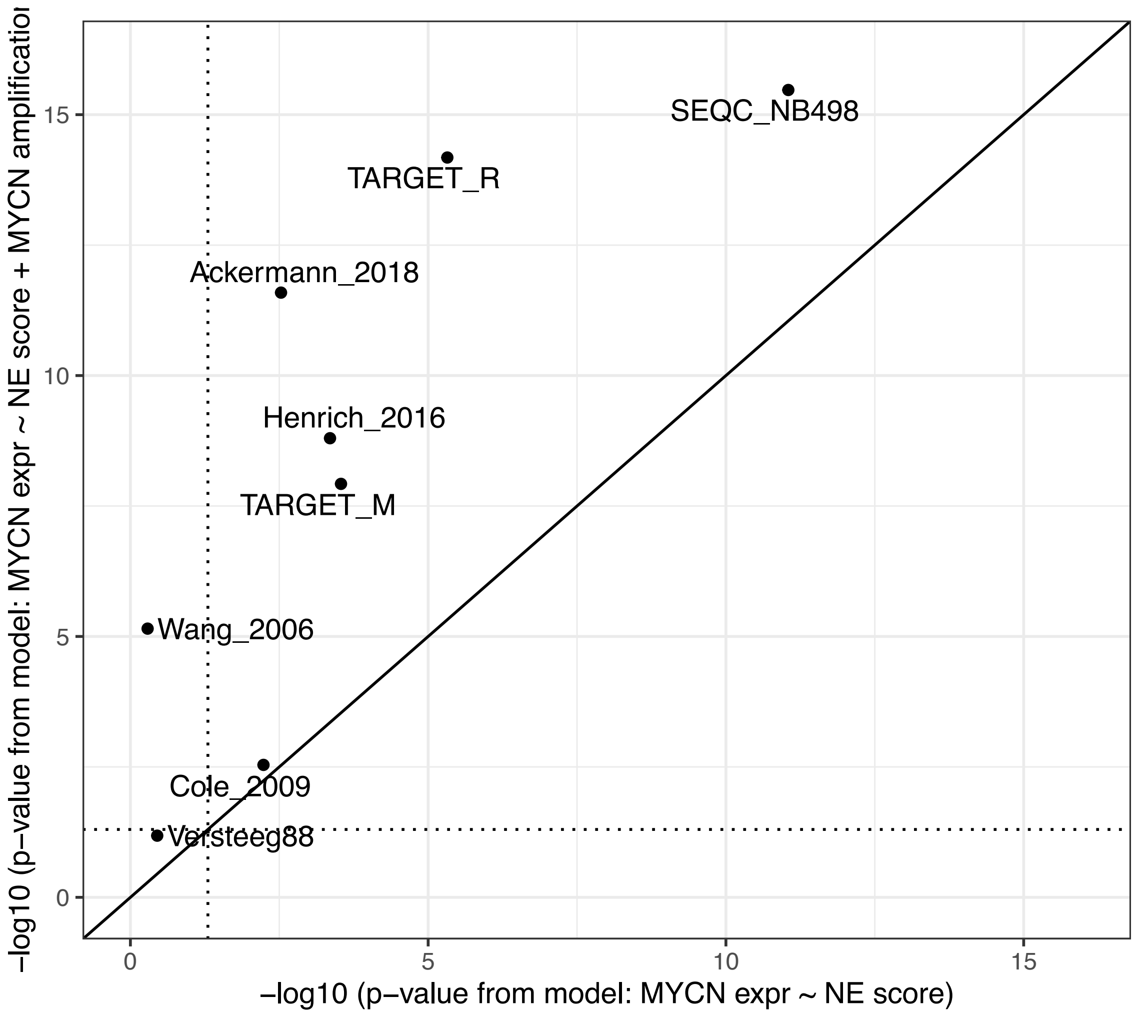


**Figure S3. Controlling for MYCN amplification status increases the statistical significance of NE score vs. MYCN expression association.**

X-axis values are p-values from a univariate linear model using NE score to predict MYCN expression, y-axis values are p-values from a multivariate linear model that included both NE score and MYCN amplification status as the predictor variables and MYCN expression as the response variable. For both models, p-values for the NE score term were extracted for comparison. Results from eight studies were included and study names were labeled on the plot. Dotted line indicates p-value = 0.05.
