# Supplementary material for: A Comparative Study of Neuroendocrine Heterogeneity in Small Cell Lung Cancer and Neuroblastoma
Source: Mol Cancer Res. 2023 May 12;21(8):795–807. doi: 10.1158/1541-7786.MCR-23-0002 (PMC10390888; doi:10.1158/1541-7786.MCR-23-0002)
Supplement: Figure S4 — SCLC vs. NBL concordance of NE score association with omics, drug sensitivity, and dependency data. [file mcr-23-0002_figure_s4_suppsf4.pdf]

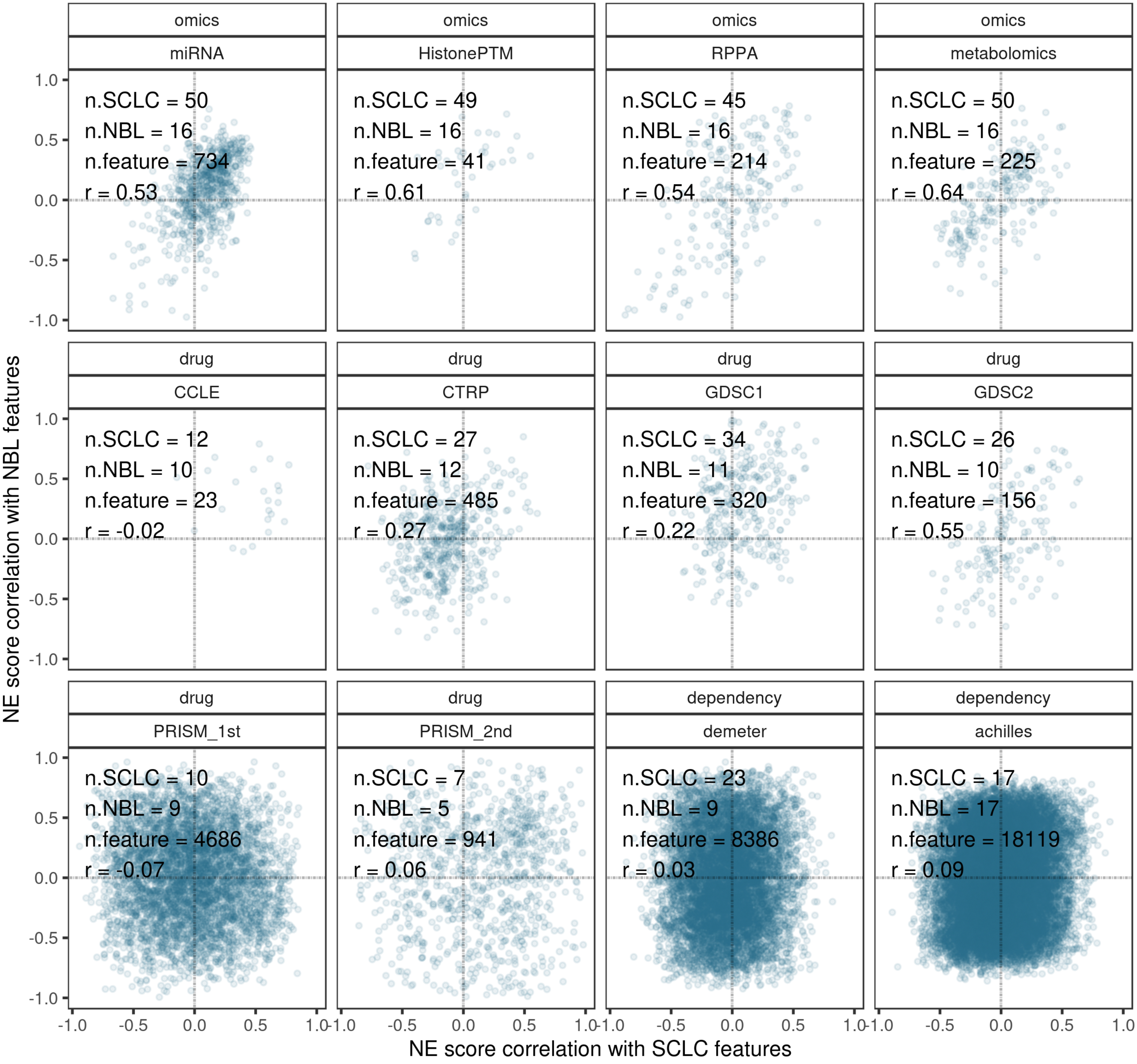

Figure S4. SCLC vs. NBL concordance of NE score association with omics, drug sensitivity, and dependency data

For each dataset, we correlated each feature with NE scores in SCLC cell lines to obtain a set of correlation coefficients (x-axis values); we also generated such values for NBL cell lines (y-axis values). Correlation from correlating SCLC results and NBL results was further conducted to summarize an overall concordance metric “r” for each dataset. Parameters printed at the upper left quadrant of each plot: n.SCLC, the median number of SCLC cell lines in correlation; n.NBL, the median number of NBL cell lines in correlation; n.feature, the number of features in the dataset; r, Pearson correlation coefficient from correlating x-axis and y-axis values, representing concordance between SCLC and NBL NE score correlations.
