# Supplementary material for: A Comparative Study of Neuroendocrine Heterogeneity in Small Cell Lung Cancer and Neuroblastoma
Source: Mol Cancer Res. 2023 May 12;21(8):795–807. doi: 10.1158/1541-7786.MCR-23-0002 (PMC10390888; doi:10.1158/1541-7786.MCR-23-0002)
Supplement: Figure S5 — A meta-analysis example that summarizes the association between MEK inhibitor resistance and NE scores. [file mcr-23-0002_figure_s5_suppsf5.pdf]

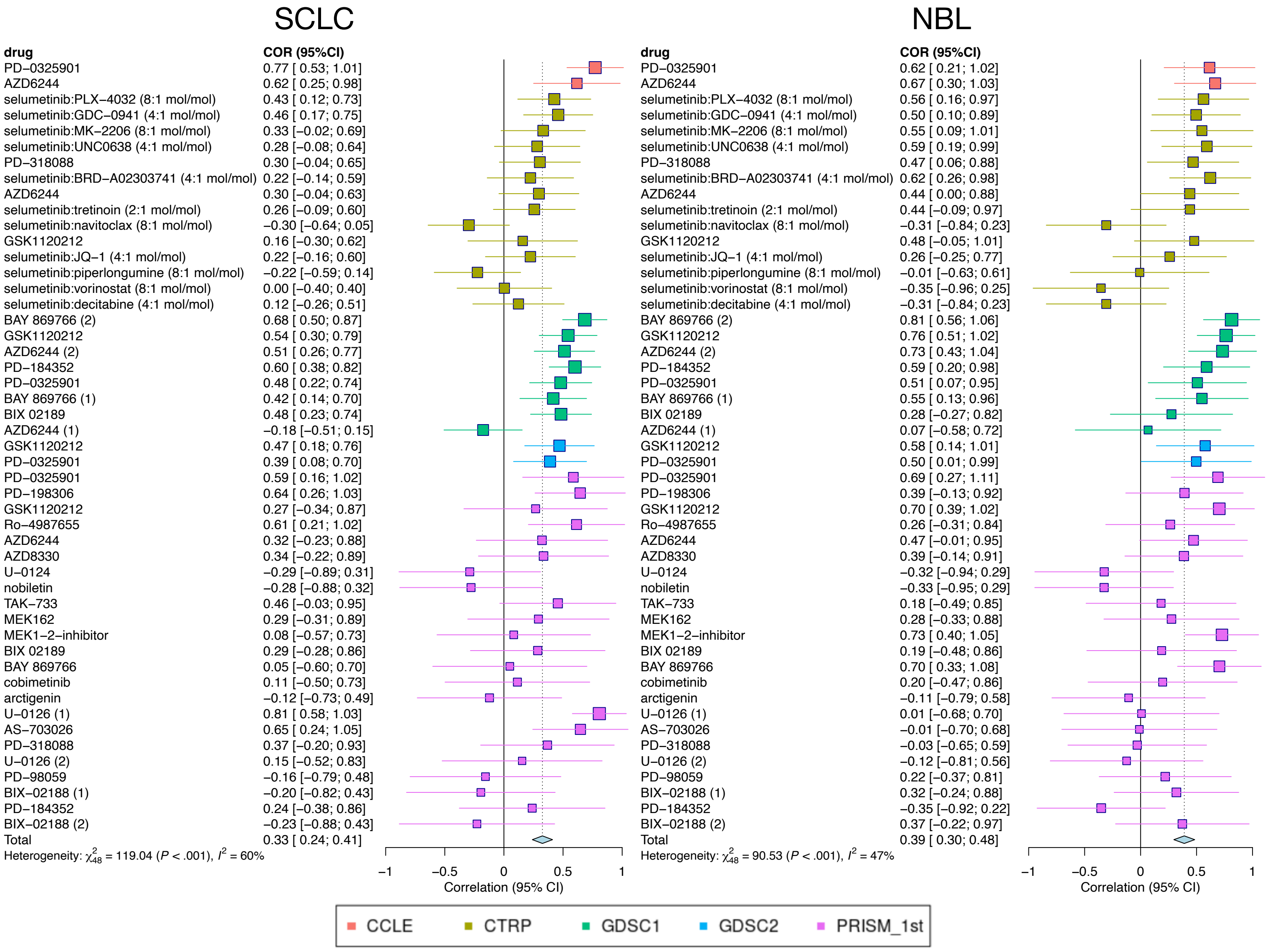

Figure S5. A meta-analysis example that summarizes the association between MEK inhibitor resistance and NE scores

Compounds that target MEK were identified from five different datasets. Correlation between therapeutic sensitivity of different MEK inhibitors and NE scores in SCLC and NBL cell lines were computed. These forest plots visualize the meta-analyses of these correlations in SCLC (left) and NBL (right). Sources of data were annotated in different colors. Datasets have been harmonized such that a positive correlation indicates cell lines with higher NE scores are more resistant.
