# Supplementary material for: A Comparative Study of Neuroendocrine Heterogeneity in Small Cell Lung Cancer and Neuroblastoma
Source: Mol Cancer Res. 2023 May 12;21(8):795–807. doi: 10.1158/1541-7786.MCR-23-0002 (PMC10390888; doi:10.1158/1541-7786.MCR-23-0002)
Supplement: Figure S6 — Examples of different and similar compound sensitivity dynamic ranges in SCLC and NBL. [file mcr-23-0002_figure_s6_suppsf6.pdf]

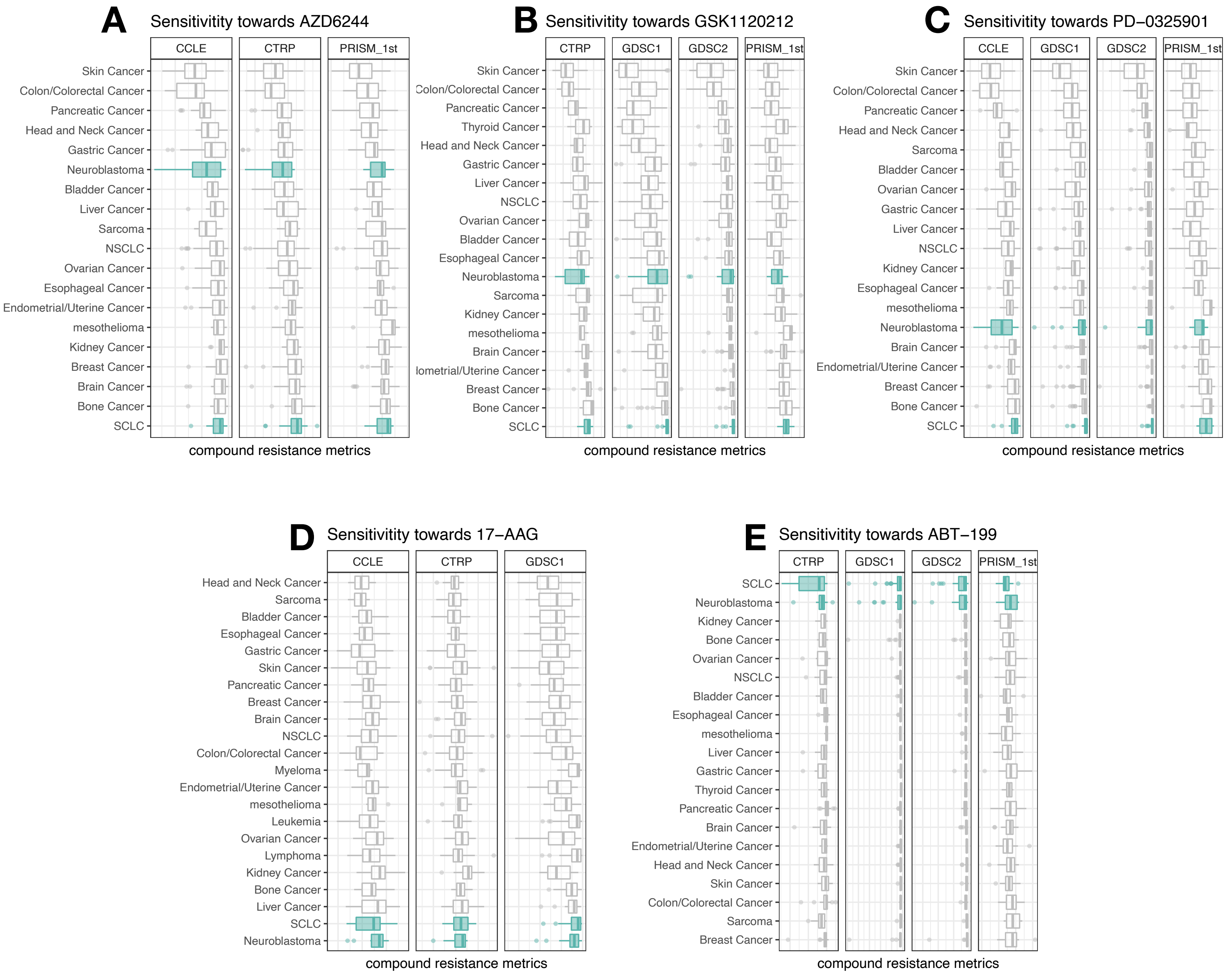

Figure S6. Examples of different and similar compound sensitivity dynamic ranges in SCLC and NBL

A-E, Distribution of compound sensitivity data by cancer lineage for selected MEK inhibitors (**A-C**), HSP90 inhibitor (**D**), and BCL inhibitor (**E**). Compound resistance metrics were z-transformed within each study. For each compound, the cancer lineages were ordered by median compound resistance values from all studies. Note that skin cancer (primarily melanoma) cell lines are most sensitive to MEK inhibitors whereas SCLC lines are most resistant to MEK inhibitors. NBL cell lines exhibit a broad distribution of sensitivity and have intermediate sensitivity to MEK inhibitors compared to the other cancer types (**A-C**). SCLC and NBL exhibited more similar compound sensitivity ranges for 17-AAG (**D**) and ABT-199 (**E**).
