# Supplementary material for: A Comparative Study of Neuroendocrine Heterogeneity in Small Cell Lung Cancer and Neuroblastoma
Source: Mol Cancer Res. 2023 May 12;21(8):795–807. doi: 10.1158/1541-7786.MCR-23-0002 (PMC10390888; doi:10.1158/1541-7786.MCR-23-0002)
Supplement: Figure S7 — Comparing NE score-associated features between SCLC and other cancer types. [file mcr-23-0002_figure_s7_suppsf7.pdf]

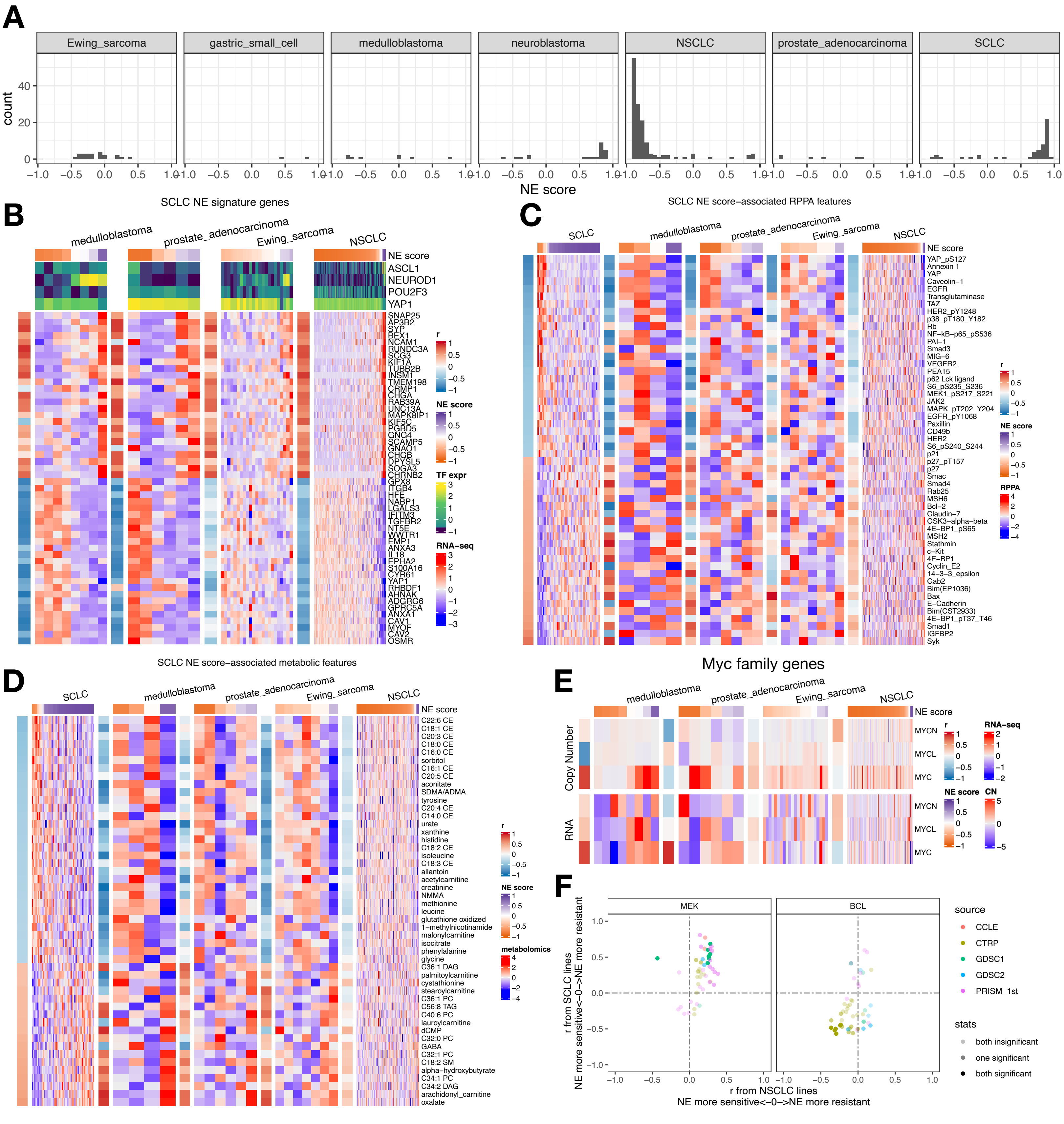

Figure S7. Comparing NE score-associated features between SCLC and other cancer types

A, Distribution of NE scores in lineage subtypes with at least two cell lines that have positive NE scores. Ewing sarcoma, medulloblastoma, NSCLC and prostate adenocarcinoma are selected for further analyses. **B**, SCLC NE signature gene expression in selected cell lines of selected cancer lineages. **C-D**, RPPA (**c**) and metabolomics (**D**) features that associate with NE scores in SCLC cell lines also associate with NE scores in cell lines of selected cancer lineages. **E**, Copy number and RNA expression of Myc family genes in cell lines of selected lineages. **F**,Correlation between NE scores and therapeutic sensitivity for drugs with selected targets. Drug sensitivity data was previously harmonized such that a higher value represents more resistance in each study. For each of the nine selected targets, all compounds with the same target were identified from multiple studies. Pearson correlation coefficient r from correlating compound data with NE scores were calculated for NSCLC lines (x-axis values) and SCLC lines (y-axis values) respectively and visualized as a scatter plot, with colors annotating the source of data, and transparency annotating the statistical significance.
